# Epidemiology of malaria and anemia in high and low malaria-endemic North-Eastern districts of India

**DOI:** 10.3389/fpubh.2022.940898

**Published:** 2022-07-28

**Authors:** Hari Shankar, Mrigendra Pal Singh, Syed Shah Areeb Hussain, Sobhan Phookan, Kuldeep Singh, Neelima Mishra

**Affiliations:** ^1^ICMR-National Institute of Malaria Research, New Delhi, India; ^2^Indian Council of Medical Research, New Delhi, India; ^3^ICMR-National Institute of Malaria Research Field Unit, Guwahati, India

**Keywords:** anemia, epidemiology, hemoglobin, low-density malaria, *Plasmodium*

## Abstract

Anemia and malaria are the two major public health problems that lead to substantial morbidity and mortality. Malaria infection destroys erythrocytes, resulting in low hemoglobin (Hb) levels known as anemia. Here we report the determinants of anemia in high and low malaria-endemic areas that would help understand which parasite densities, age, and gender-associated low Hb levels. Therefore, a cross-sectional mass survey (*n* = 8,233) was conducted to screen anemia and malaria in high and low malaria-endemic districts (HMED and LMED) of North-East India. Axillary body temperature was measured using a digital thermometer. The prevalence of anemia was found to be 55.3% (4,547/8,233), of which 45.1% had mild (2,049/4,547), 52.1% moderate (2,367/4,547) and 2.9% had severe anemia (131/4,547). Among anemic, 70.8% (3,219/4,547) resided in LMED and the rest in HMED. The median age of the anemic population was 12 years (IQR: 7–30). Overall, malaria positivity was 8.9% (734/8,233), of which HMED shared 79.6% (584/734) and LMED 20.4% (150/734) malaria burden. The village-wise malaria frequency was concordant to asymptomatic malaria (10–20%), which showed that apparently all of the malaria cases were asymptomatic in HMED. LMED population had significantly lower Hb than HMED [standardized beta (β) = −0.067, *p* < 0.0001] and low-density *Plasmodium* infections had higher Hb levels than high-density infections (β = 0.113; *p* = 0.031). Women of reproductive age had higher odds for malaria (OR: 1.42; 95% CI: 1.00–2.05; *p* = 0.04). Females (β = −0.193; *p* < 0.0001) and febrile individuals (β = −0.029; *p* = 0.008) have shown lower Hb levels, but malaria positivity did not show any effect on Hb. Young children and women of reproductive age are prone to anemia and malaria. Although there was no relation between malaria with the occurrence of anemia, we found low-density *Plasmodium* infections, female gender, and LMED were potential determinants of Hb.

## Introduction

Anemia is a significant public health concern; approximately 2.36 billion people worldwide are affected by this condition (1). Nutritional deficiencies are the most common cause of anemia; however, other reasons include hemolysis due to congenital or acquired blood disorders, blood loss, hemoglobinopathies, and infections such as helminthiasis, malaria, tuberculosis, HIV, etc. (2). The present study was conducted in two districts of North-East India, namely East Garo Hills (Area: 2,603 Sq. Km with populations of 317,917) of Meghalaya state and Udalguri (Area: 2,012 Sq. Km with populations of 831,668) of Assam state. The average literacy rate of districts East Garo Hills and Udalguri was 73.9% and 65.4%, respectively, according to the Census Population 2011 data of India (3, 4). Geographically, the district of East Garo Hills lies at 25076' N latitude, 90068' E longitude, and 600 m altitude with an 87.9% forest-covered area (5). In contrast, district Udalguri lies at 26075' N latitude, 92,010' E longitude, and 180 m altitude with a 20.5% forest-covered area (6). The economy of both districts is mainly dependent on the agriculture sector. In East Garo Hills district, the Gross District Domestic Product (GDDP) during 2007-2008 was INR 5.752 billion, and the Per Capita Income was INR 18,771 (5); whereas, district Udalguri had the GDDP during 2009-2010 of INR 21.510 billion, and the Per Capita Income was INR 16,996 (6). The healthcare indicators data of the districts revealed a better health profile of the populations from East Garo Hills compared to the Udalguri population. The indicators such as antenatal health check-ups during the first trimester (59.2% in East Garo Hills *vs*. 50.1% in Udalguri), women with BMI below standard, i.e., <18.5 Kg/m^2^ (8.5% in East Garo Hills *vs*. 16.1% in Udalguri), percentage of children under 3 years breastfed within 1 h of delivery (48% in East Garo Hills *vs*. 46.5% in Udalguri), stunting (39.7% in East Garo Hills vs. 33.8% in Udalguri), wasting (20.1% in East Garo Hills vs. 21.3% in Udalguri), severe wasting (6.2% in East Garo Hills vs. 11.6% in Udalguri) and underweight (26.5% in East Garo Hills *vs*. 32.5% in Udalguri) status of the children below 5 years of age (7, 8). *Plasmodium falciparum* was the major malaria parasite in both districts and contributed to more than 90% of the total malaria cases.

In India, the data from National Family Health Survey-4 (NFHS-4) conducted in 2014-2015 revealed that children up to 5 years (58.6%), 53.2% non-pregnant, 50.4% pregnant women, and 22.7% of men from 15–49 years of age were suffering from anemia (9). According to NFHS-4, district East Garo Hills of Meghalaya state had a higher anemia prevalence than district Udalguri of Assam state; whereas, the anemia situation is completely reversed in the latest NFHS-5 report showing a very high prevalence of anemia in district Udalguri (children (6–59 months): 77.6%, women (15–49 years): 81.5%, women (15–19 years): 72.6%) (8) than district East Garo Hills (children (6–59 months): 28%, women (15–49 years): 50.9%, women (15–19 years): 55.7%) (7). Malaria, on other hand, is another big challenge for the public health research community. In 2019, it resulted in 229 million new infections and nearly 0.409 million deaths in 87 countries across the globe (2). Due to the continuous efforts of the Indian Government, though there has been a constant decline in malaria cases since 2015, there was still a considerable number of malaria cases (0.34 million) reported in the year 2019 (10). In malaria-endemic regions, anemia severity is one of the significant consequences of malaria, and young children are the ones who withstand the penalties (11–13); as they grow up, they mostly become asymptomatic *Plasmodium* parasite carriers. In high transmission areas, the risk of anemia due to malaria is higher in young children and infants. In contrast, low transmission regions reflect more symptomatic malaria and associated anemia among all age groups, but the chances of anemia are comparatively higher in children and pregnant women (14). In addition, it has been reported that moderate-to-severe anemia may be used as a proxy indicator of reduced malaria burden in an area of stable malaria transmission (15). The impact of malaria interventional tools such as insecticide-treated bed nets, indoor residual spraying, and malaria chemoprevention was more pronounced on anemia, even better than the indicators like mortality/ malaria parasitemia estimates or incidence of clinical malaria (14, 16).

An epidemiological assessment of malaria cases in district Udalguri from the year 2008 to 2018 showed a drastic decline in annual parasite incidence (API) from 14.5 to 2.6 cases per 1,000 population. The transmission of malaria in this district starts in April, and the peak is observed in June/ July. In 2018, *P. falciparum* was the predominant species that caused the majority of malaria infections (>80), and the rest of the malaria cases were due to *P. vivax* (17). Contrary to this, malaria in Meghalaya state is persistent and perennial, with seasonal spikes seen in the monsoon, which peaks from May to July and sometimes extends up to September to November. *P. falciparum* is this region's predominant parasite species responsible for malaria cases (18). A systematic review and analysis of malaria data from the National Vector Borne Disease Control Programme revealed that district East Garo Hills had the highest API (>10) in the year 2016 among all districts of Meghalaya state. The study also reported a sharp decline in malaria after the distribution of long-lasting insecticide-treated bed nets (LLINs) in 2016. This decline continued in the year 2017 (84.7% reduction in malaria relative to 2015 cases). In contrast, the proportion of individuals >15 years of age with malaria increased from 42.1% in 2015 to 47.2% in the year 2017 (*p* < 0.001) (19). Our previous findings evidenced the presence of asymptomatic and low-density *Plasmodium* infections in district East Garo Hills, Meghalaya state, and Udalguri, Assam state (20). However, there is a lack of information about the anemia profile of this population and its relationship with low-density infections and other determinants of anemia. Therefore, we conducted mass screening for anemia and malaria in the villages of high and low-malaria endemic districts in two North-Eastern states of India. This study would help elucidate the anemia and malaria situation in this region and also estimate the determinants of anemia.

## Materials and methods

**Figure 1:**
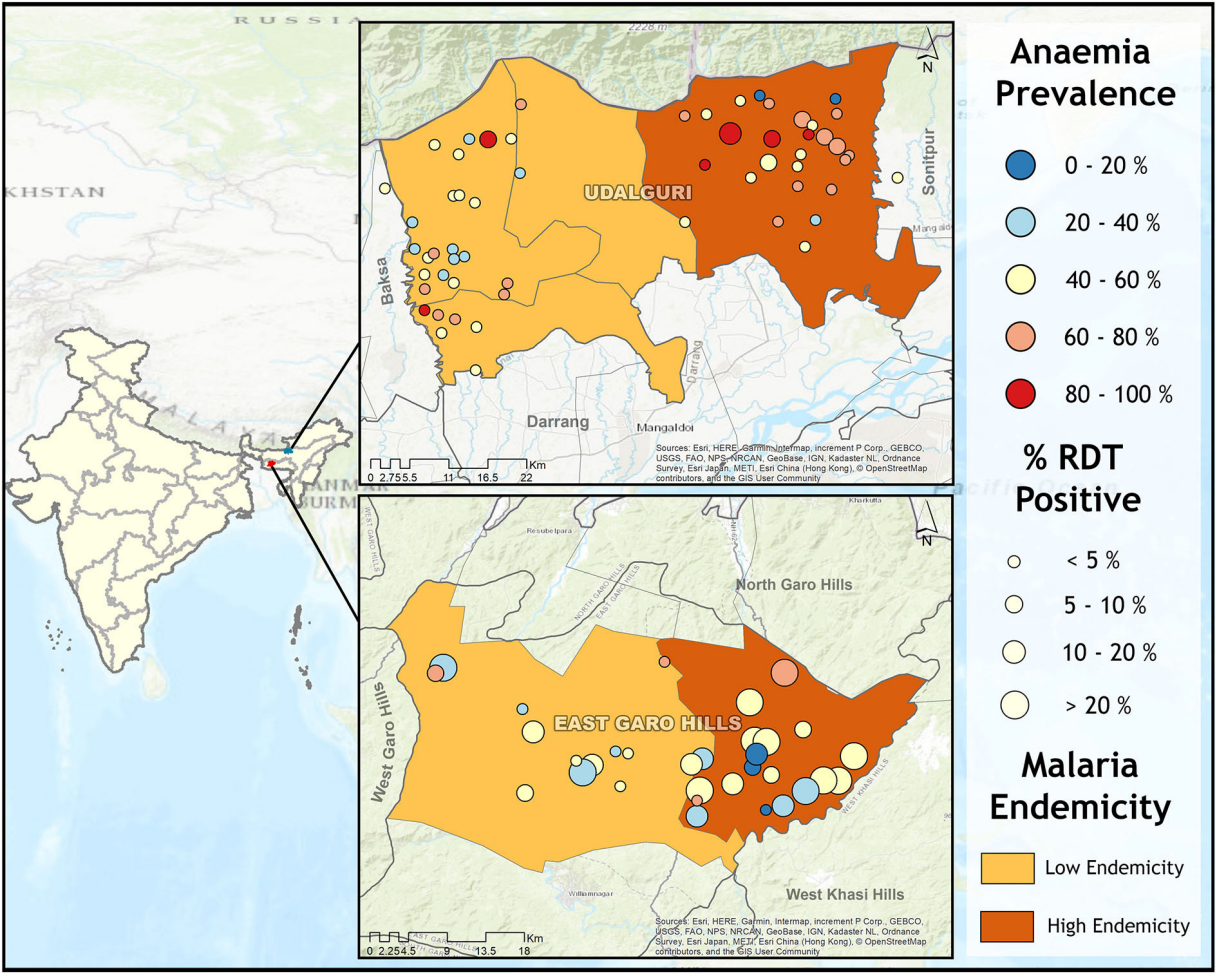
Map showing anemia and malaria prevalence in study villages from high (East Garo Hills) and low malaria-endemic districts (Udalguri). Each district was further categorized as low endemic (light brown) and high endemic (dark brown) based on previous years' Block Primary Health Centers (PHCs) data. Circle size represents malaria prevalence and the circle color represents anemia prevalence.

We conducted a community-based mass screening for malaria and anemia in villages of two North-Eastern districts Udalguri, Assam state, and East Garo Hills, Meghalaya state in India. Based on the previous 5 years of malaria epidemiological data, it was observed that Udalguri district was a low malaria-endemic district and East Garo Hills district was a high malaria-endemic district. In Indian districts, all the villages cover under primary health centers (PHCs). Two PHCs from each district were randomly selected (one with high annual parasite incidence (API) recognized as a high-endemic PHC, and another with low API as a low-endemic PHC) based on the past 5 years of malaria epidemiological data of PHCs under the study districts. Further, 10 villages from each PHC were selected using a probability proportional to size (PPS) sampling technique. In a situation where the required sample from the selected village was not available, the shortfall was covered from adjacent villages. The map in Figure 1 shows the malaria endemicity of the selected PHCs based on color coding. The low endemic PHC is shown in light brown color and the dark brown region depicts high endemic PHC (Figure 1). This mass survey was conducted from February to April 2017. For better participation of the community, accredited social and health activists (ASHAs) communicated the objectives of the survey and the program schedule in the selected villages 1 day before the visit. The individuals who were willing to participate in the study were screened for anemia and malaria, irrespective of clinical signs and symptoms related to malaria or anemia. The aims and objectives of the study were informed to the participants and informed consent/ assent forms were obtained from each participant prior to the enrolment in this study. Those participants who had the presence of one or more danger signs of severe malaria, severe malnutrition, contraindications related to the antimalarial drugs used (especially history of allergies), and pregnant women were not enrolled in the study. A total of 8,233 individuals irrespective of age and gender were finger-pricked for screening of anemia by measuring hemoglobin (Hb) concentration using HemoCue Hb 201 analyzer (HemoCue, Angelholm, Sweden). Simultaneously, malaria screening was performed using rapid diagnostic tests (RDTs) in 8,232 individuals, light microscopy (*n* = 7,754), and polymerase chain reaction (PCR) (*n* = 3,100). Out of 8,233, 5,525 participants were from the district Udalguri (low malaria-endemic) and 2,708 were recruited from East Garo Hills district (high malaria-endemic). There were 57% (4,680/8,233) female participants in the study and the rest were male. The RDT kits used in the study were SD Bioline malaria Ag Pf/Pv, Haryana, India. Giemsa staining was used for microscopic examination of slides to detect the presence of *Plasmodium* species. Dried blood spots (DBS) were prepared on Whatman filter paper, grade 1 (Merck, Germany, United Kingdom) for identification of *Plasmodium* parasite species using PCR. Axillary body temperature was recorded using a digital thermometer and a body temperature of ≥ 37.5°C at the time of examination was defined as febrile. The proportion of afebrile individuals in the study population was 92.4% (7,606/8,233) and febrile illness was found in 7.6% (627/8,233) participants. Afebrile participants detected with *Plasmodium* parasite through RDT were defined as asymptomatic malaria cases. Low-density *Plasmodium* infections were defined as those malaria cases which were detected by PCR but undetected by microscopy and/or RDT. An individual was considered “malaria positive” only when detected with *Plasmodium* parasite either by light microscopy, RDT or by PCR. Anemia was defined according to the age-group as classified by WHO (21). The outcome variable in this study was anemia and the exposure variables were low density *Plasmodium* infection, study districts, gender, febrile illness and malaria positivity. The village wise demographics and the prevalence of malaria (based on RDT tests) and anemia (based on Hb values) was calculated as percentages for the selected villages, blocks and districts (Supplementary Table 1). The villages selected for the study were identified on Google Earth and the respective coordinates of each of the villages were plotted in ArcGIS 10.2.1 software to represent the village-wise prevalence of malaria, asymptomatic cases of malaria, and anemia severity on maps.

Thick and thin blood smears were prepared at the study site and transported daily to the local laboratory, where fixation of thin blood smears was performed with methanol followed by staining of both thick and thin smears with 3% Giemsa stain solution for 30 min. All the slides were examined under oil emersion (10 × 100 magnification) by experienced microscopists. A blood slide was considered negative if no *Plasmodium* parasites were found after examining 100 microscopy fields. For all positive slides, identification of *Plasmodium* species was performed. All the microscopists were kept blinded to RDT results and later the discordant results (RDT^+ve^ Microscopy^−*ve*^ and RDT^−*ve*^ Microscopy^+ve^) were re-examined by another experienced microscopist and/ or a WHO certified microscopist who was not aware of previous microscopy/ RDT results. The final microscopy results were considered positive or negative depending upon the quality check results of the microscopy.

The *Plasmodium* species identification using PCR was done on DBS prepared at ambient temperature and sealed in zipped plastic bags containing desiccant and stored at−20°C until analysis. To perform the diagnostic PCR, DNA was isolated from DBS using the QIAamp Blood Mini Kit (QIAGEN, United States) according to the manufacturer's protocol. The protocol followed in this study to identify *Plasmodium* species using PCR is described elsewhere, which was based on targeting coding sequences of the small subunit of ribosomal RNA specific to the parasite species. The limit of detection ranged from 1 to 10 parasites/μl of blood. The details of the primer sequences used in this study have been described previously (22). Briefly, 25-μl reaction mixtures consisted of 12.5 μl DreamTaq Green PCR mix, 0.4 μM of each primer (10 μM stock), 2 μl of DNA, and sterile water up to 25 μl. All PCR amplifications were carried out in an Applied Biosystems (Waltham, MA, United States) thermocycler as follows: 5 min at 95°C, followed by 35 cycles of 30 s at 95°C, 30 s at 56°C, and 1 min at 72°C, with a final extension of 7 min at 72°C. PCR amplifications were analyzed on 2% agarose gels prepared with 0.5 × Trisborate-ethylenediaminetetraacetic acid buffer in the presence of ethidium bromide. After obtaining the PCR results, the discordant samples (RDT^+ve^ Microscopic^+ve^ PCR^−*ve*^ and RDT^−*ve*^ Microscopic^−*ve*^ PCR^+ve^) were re-confirmed using light microscopy and PCR.

### Statistical analysis and data management

The data were entered into Microsoft Excel 2007 worksheet and cross-checked for typographical errors. Further, lists of quality checks have been applied in order to ensure the quality of data. Non–numerical categorical variables were coded numerically and frequency with percentage distribution was tabulated and Pearson's Chi square or Fisher's exact test was applied for statistical comparison of 2 × 2 contingency tables as appropriate. Standardized beta coefficient (unadjusted and adjusted) was calculated using univariate and multivariate regression model to find out determinants of Hb concentration in study population. A univariate linear regression was performed to calculate unadjusted standardized beta coefficient; whereas, adjusted standardized beta coefficient was calculated using multivariate regression model in which the effect of one variable on another was studied in the presence of other covariates. Logistic regression analysis was applied to calculate the Odds ratio (OR) with 95% confidence intervals (CI) to understand the likelihood of anemia severity among females with reference to males. Critical value for statistical significance was considered at alpha 0.05. Data were analyzed using statistical software R 3.4.3 for Windows (R Project for Statistical Computing).

## Results

**Figure 2:**
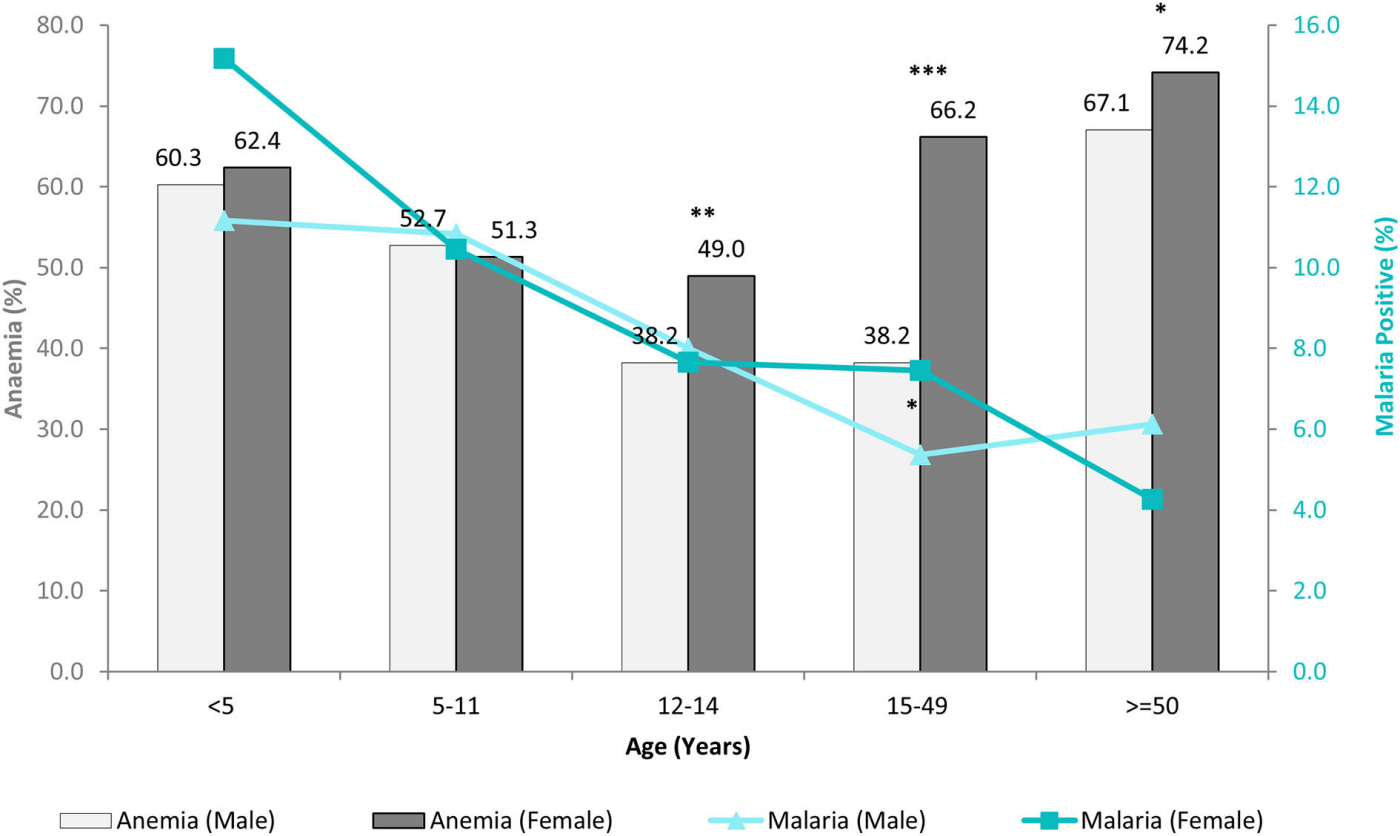
Age and sex-wise distribution of anemia and malaria cases. (Significant difference between male and female *p < 0.05; **p = 0.001; ***p= 0.000).

In this study, overall malaria positivity irrespective of any diagnostic method used was 8.9% (734/8,233), of which high endemic district shared 79.6% (584/734) and low endemic 20.4% (150/734) of malaria burden. Figure 2 showed age and sex-wise distribution of anemia and malaria cases which revealed that females of the pubic age group had significantly higher anemia 49% (262/535) than males 38.2% (167/437) (χ^2^ = 11.29; *p* = 0.001), whereas malaria positivity was more or less similar in this age-group. This difference was more pronounced among women of reproductive age, where anemia prevalence was 66.2% (1,179/1,782) in females and 38.2% (335/876) in males (χ^2^ = 186.74; *p* = 0.000). Women of reproductive age-group 7.5% (133/1,782) were also found to have higher odds for malaria positivity (Odds ratio (OR) 1.42 at 95% confidence interval (CI): 1.00–2.05; *p* = 0.04) than men 5.4% (47/876). Malaria positivity in this population declined with the advancement of age, i.e., young children (<5 years) had the highest malaria positivity (13.2%) compared to adults (Figure 2). In this study, low-density *Plasmodium* infections were found in 38.2% (139/364) cases, of which 49.6% (69/139) were anemic. Among low-density infections, 56.8% (79/139) cases were from high malaria-endemic district and 43.2% (60/139) from the low malaria-endemic district. Overall asymptomatic *Plasmodium* infections were 8.3% (635/7,606), of which the proportion of anemic cases was 51.8% (329/635). Among asymptomatic cases, 77.5% (492/635) cases were from high and, 22.5% (143/635) were from the low malaria-endemic district.

**Figure 3:**
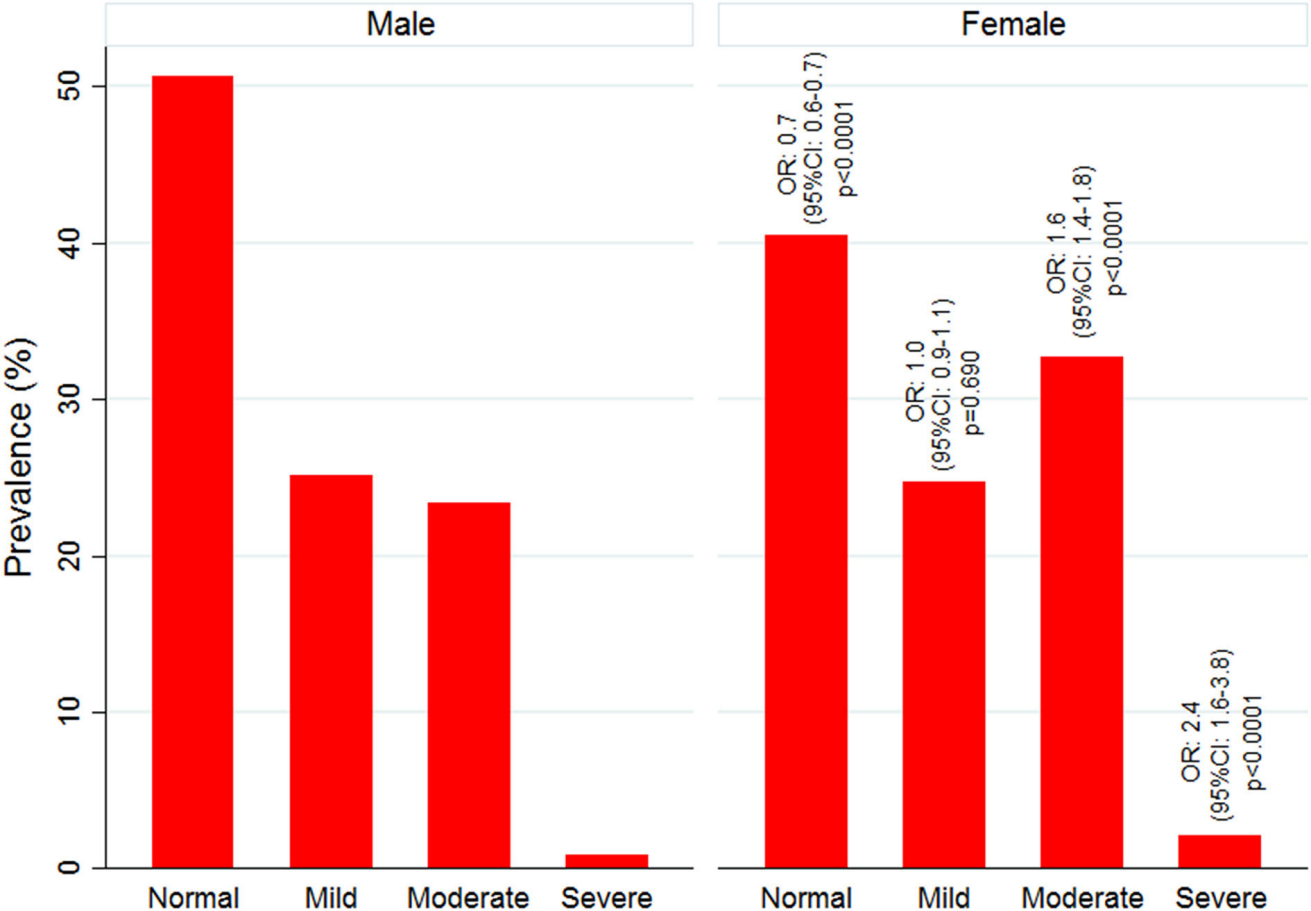
Anemia grading and its likelihood among male and female participants.

**Figure 4:**
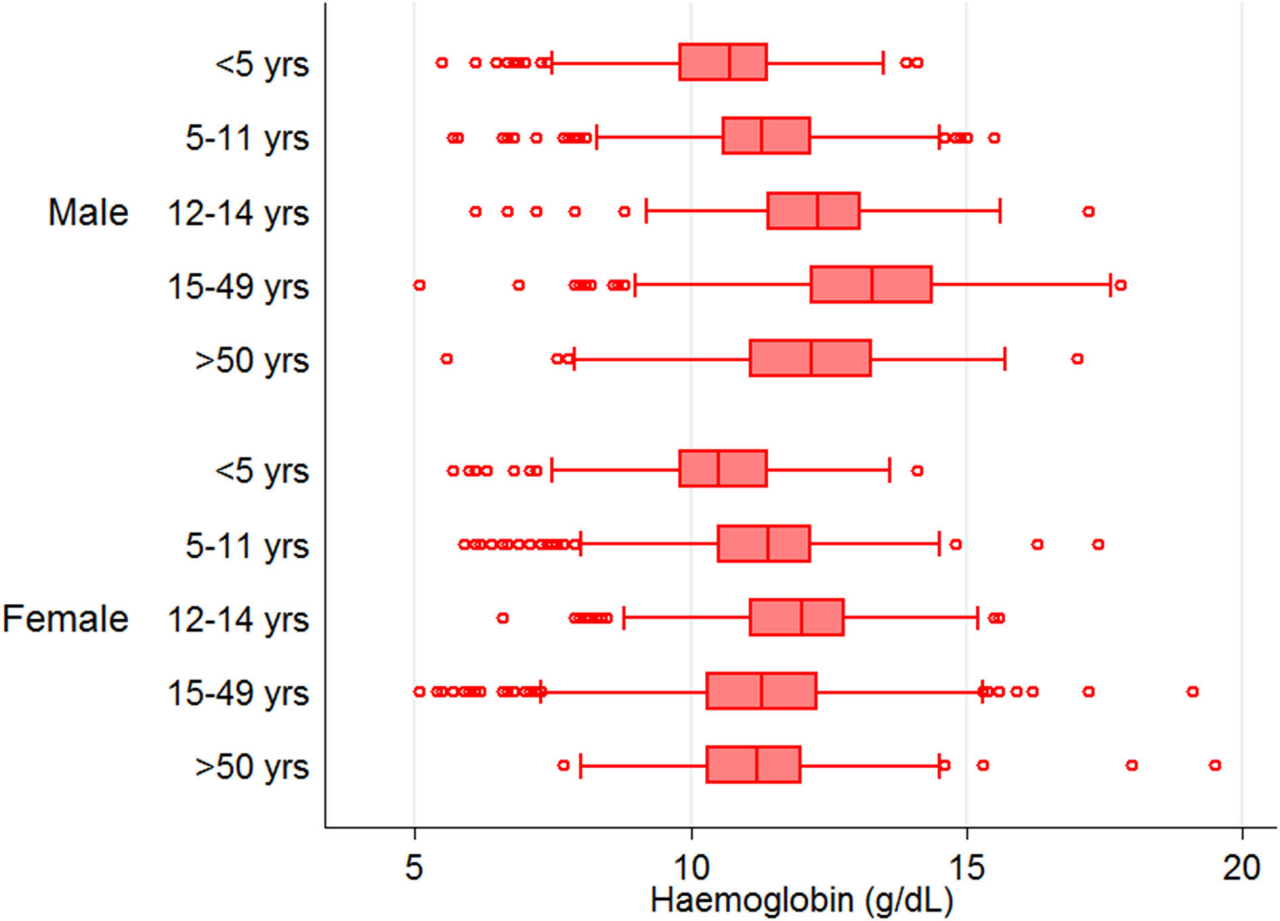
Age category and sex-wise distribution of hemoglobin levels.

Mean Hb concentration in the study population was 11.5 g/ dl at 95% CI: 10.6–12.5, and anemia prevalence was 55.3% (4,547/8,233). Among anemic individuals, 45.1% were mild (2,049/4,547), 52.1% were moderate (2,367/4,547), and 2.9% were severe anemic (131/4,547). Of anemic population, 70.8% (3,219/4,547) belong to low malaria-endemic district, and the rest are from high endemic district. The study map shows the prevalence of anemia and malaria RDT positivity in the investigated villages (Figure 1). Anemia severity based on the sex of participants showed that nearly 50% of males and 60% of females were anemic. Females had 30% fewer chances of being normal than males (OR: 0.7; 95% CI: 0.6–0.7; *p* < 0.0001). Females also had higher odds for moderate anemia (OR: 1.6; 95% CI: 1.4–1.8; *p* < 0.0001) and severe anemia (OR: 2.4; 95% CI: 1.6–3.8; *p* < 0.0001) in comparison to males. Among males, the frequency of mild anemia was highest (25.1%), whereas the majority of females had moderate anemia (32.7%), and 2.1% were severe anemic (Figure 3). The overall median age of anemic population was 12 years with an interquartile range (IQR) of 7–30 years, where anemic males were 9 years (IQR 6–22) and anemic females were 16 years (IQR 8–34) of age. In the high malaria-endemic district, the median age of anemic individuals was 11 years (IQR 5–30) having anemic males were 7 years (IQR 4–19), and anemic females were 18 years (IQR 6–30) age. However, the median age of anemic individuals in the low malaria-endemic district was 12 years (IQR 8–32), of which males were 10 years (IQR 7–25), and females were 16 years (IQR 8–35) of age. The results suggest that irrespective of the gender, median Hb levels were lowest in <5 years of age children, and there was a progressive increase in Hb levels up to 50 years in males, and up to pubic age, i.e., 12–14 years in females, and declined after that (Figure 4).

**Figure 5:**
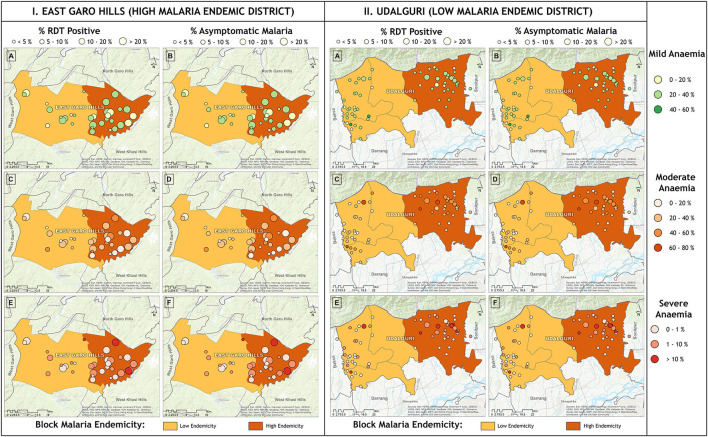
Village wise anemia severity against prevalence of malaria (RDT positive) and asymptomatic malaria cases in **(I)** high malaria endemic district and **(II)** low malaria endemic district. Horizontal panel shows frequency of anemia severity and vertical panel shows malaria.

The village-wise frequency of anemia severity was plotted against the prevalence of malaria cases (RDT positives) and asymptomatic malaria cases. In high endemic district severe anemia prevalence was below 5% in most villages except in two where prevalence was 10–20%, and moderate and mild anemia was between 20–40% in most villages. In the high endemic district, villages under high endemic PHC (dark brown region) showed high malaria positivity. Most villages, except a few, had asymptomatic *Plasmodium* infections between 10-20% or above (Figure 5.I.). Nearly half of the studied villages under low endemic PHC (light brown region) had 10-20% cases of asymptomatic malaria, but anemia prevalence was similar to villages under high endemic PHC. Being a low malaria-endemic district (Figure 5.II.). RDT positivity and asymptomatic *Plasmodium* infections were similar (<5%) in villages under high endemic PHC, and apparently, no malaria (only 1 RDT positive) was found in low endemic PHC. However, mild anemia was nearly 20–40% in most villages, frequency of moderate anemia was 40–60% in villages under high endemic PHC and 20–40% in villages under low endemic PHC. In high endemic PHC, the frequency of severe anemia cases was above 10% in five, and between 1–10% in 11 out of 30 villages; whereas out of 31 villages under low endemic PHC, two villages had severe anemia >10% and nine villages had severe anemia ranged between 1–10% (Figure 5.II.).

Hb concentration (mean ± sd) in high and low malaria-endemic districts was estimated at 11.7 ± 1.7 and 11.5 ± 1.6 g/dl, respectively. The analysis revealed that mean Hb concentration was significantly lower in low as compared to the high malaria-endemic districts [standardized beta (β) = −0.067; *p* < 0.0001]. Low-density *Plasmodium* infections had significantly higher Hb concentration in comparison to high density infections (mean ± sd: 11.8 ± 1.5 *vs*. 11.5 ± 1.5 g/dl, β = 0.113; *p* = 0.031). Gender female in comparison to male (11.3 ± 1.5 *vs*. 11.9 ± 1.7 g/dl, β = −0.193; *p* < 0.0001) and febrile in comparison to afebrile subjects (11.4 ± 1.6 *vs*. 11.6 ± 1.6 g/dl, β = −0.029; *p* = 0.008) had a lower concentration of Hb, but there was no effect of malaria positivity on mean Hb concentration of study participants (Table 1). After adjusting the effects of the determinants, it was observed that the area of residence, i.e., districts, parasite density, and sex of the study subjects, were the potential significant determinants of Hb concentration (Table 1).

**Table 1:** Univariate and multivariate regression model to find out determinants of Hb concentration.

**Determinants**	Hemoglobin concentration (g/dl)*	**Unadjusted**		**Adjusted**	
		**standardized beta coefficient**	***p*** **value**	**standardized beta coefficient**	***p*** **value**
**District**					
High malaria-endemic (*n* = 2,708)	11.7 ± 1.7	0 (Reference)			
Low malaria-endemic (*n* = 5,525)	11.5 ± 1.6	−0.067	<0.0001	−0.153	0.005
**Low density malaria**					
No (*n* = 225)	11.5 ± 1.5	0 (Reference)			
Yes (*n* = 139)	11.8 ± 1.5	0.113	0.031	0.177	0.001
**Sex**					
Male (*n* = 3,537)	11.9 ± 1.7	0 (Reference)			
Female (*n* = 4,680)	11.3 ± 1.5	−0.193	<0.0001	−0.138	0.008
**Fever**					
Afebrile (*n* = 7,606)	11.6 ± 1.6	0 (Reference)			
Febrile (*n* = 627)	11.4 ± 1.6	−0.029	0.008	0.016	0.753
**Malaria**					
Negative (*n* = 7,499)	11.5 ± 1.6	0 (Reference)			
Positive (*n* = 734)	11.5 ± 1.6	−0.000	0.981	Omitted	

*Values expressed in mean ± sd.

## Discussion

This study presents the prevalence of anemia and malaria in two (one high: East Garo Hills, Meghalaya, and one low malaria-endemic: Udalguri, Assam) north-eastern districts of India. We found an 11.5 g/dl mean Hb concentration in the study population, and more than one-half of the study population was anemic, of which the majority was moderate anemic. Previous studies have shown nearly 60% anemia prevalence in the tribal people of Assam (23) and around 47% in Meghalaya (24). Of anemic population, almost 71% belonged to district Udalguri and 29% to East Garo Hills. Our result demonstrates that the age-wise distribution of anemia followed a U-shaped curve (Figure 2). Figure 2 showed that anemia prevalence was higher in younger age groups, declined up to the pubic age group i.e., 12–14 years, and increased in an age-dependent manner. The results suggest that the pubic age group was the least susceptible group for anemia, and males had a significantly lower prevalence than females. A recent study from western China also supports our observations where they reported anemia as a mild public health problem among pubic age adolescents residing in west China (25).

On the other hand, malaria positivity was inversely proportional to the age, i.e., malaria positivity declined with the advancement of age (Figure 2). Despite having high malaria positivity and low-density *Plasmodium* infections in the high malaria-endemic district (East Garo Hills), anemia prevalence was low compared with the low malaria-endemic district (Udalguri). Our findings were contrary to anemia estimates reported in NFHS-4 (2015-2016) but similar to the recent NFHS-5 (2019-2020) data that reported higher anemia prevalence in district Udalguri, Assam than East Garo Hills, Meghalaya (26, 27). There are several factors other than malaria, such as low dietary intake of iron, micronutrient deficiencies, phytate-rich diet, helminthic infections, intestinal bleeding, worms, food habits, smoking, poor bioavailability, socio-economic status, sanitation, gravidity, and parity status of women, hemoglobinopathies, etc. that may affect Hb status and anemia prevalence (28). In this study, the low malaria-endemic district i.e., Udalguri had a majority of people belonging to low socioeconomic status, labor class that works in tea estates on daily wages and consume a high quantity of tea that inhibits dietary iron absorption (29) with a high prevalence of Hb S, E and β-thalassemia disease (30–32). We found that the lowest Hb concentration was present in children below 5 years of age. The factors such as fecundity of mother, multiple siblings, childbearing age of mother, and their nutrient intakes are reported to be associated with anemia in children (24). In addition, highest malaria positivity was found in the youngest age group (Figure 2), which might be one of the potential reasons for the lowest Hb levels and high anemia prevalence in children below 5 years of age. Hemoglobin concentrations were maintained till pubic age but declined in reproductive age-group women due to periodic blood loss during menstruation, repeated childbearing, lactation / breastfeeding, and irregular iron-folic acid intake (33). We observed that women of reproductive age are the most susceptible age group with significant anemia and higher chances for malaria compared to men. A higher risk of malaria in females might be because, in these study districts, many women work for their daily livelihood either by farming or by working in tea gardens or as laborers, thereby exposed to mosquito bites.

Although East Garo Hills (high malaria-endemic) district had higher malaria positivity and low-density infections, there was a better Hb profile of residents from this district. This might be due to their dietary habits, mainly consuming meat products (34), a direct source of hem iron in their meals. Further, it has been reported that high parasitaemia is associated with increased prevalence of anemia and reduced Hb concentration (35). In our study, we found higher Hb concentration in low-density *Plasmodium* infections, and most of such infections were present in high malaria-endemic district, i.e., East Garo Hills. Age and sex are essential determinants for anemia severity, and we found lower Hb concentration in females than males and in febrile compared to afebrile participants. Many confounding factors affect anemia status, including bacterial / viral / helminthic infections, genetic abnormalities / mutations in the population, micronutrient deficiencies, dietary / lifestyle habits, parity and gravidity of women, socio-economic status, etc. These confounders were not examined, which is the main limitation of this study.

Our study suggests no relation between malaria positivity with anemia prevalence in the study districts. The low malaria-endemic district had higher anemia but lower malaria positivity than the high endemic district. We observed that younger children and females from reproductive age groups were most susceptible to anemia and malaria. Low-parasite density, low malaria endemicity, and female residents were the potential determinants influencing Hb concentration in this study population.

## Data availability statement

The original contributions presented in the study are included in the article/Supplementary material, further inquiries can be directed to the corresponding author.

## Ethics statement

The studies involving human participants were reviewed and approved by Institutional Ethics Committee of National Institute of Malaria Research. Written informed consent to participate in this study was provided by the participants' legal guardian/next of kin.

## Author contributions

HS contributed substantially in data collection, data analysis, interpretation, and drafting of manuscript. MS: data cleaning, statistical analysis, and interpretation. SH: mapping village wise data and compilation of results. SP: study design, data collection, and study management. KS: data collection and logistic arrangements at the study sites. NM: conception and design of the study, study management, and editing of the manuscript. All authors contributed to the article and approved the submitted version.

## Funding

This study was funded by Indian Council of Medical Research, New Delhi, India vide grant no. NER/55/2015-ECD-I.

## Conflict of interest

The authors declare that the research was conducted in the absence of any commercial or financial relationships that could be construed as a potential conflict of interest.

## Publisher's note

All claims expressed in this article are solely those of the authors and do not necessarily represent those of their affiliated organizations, or those of the publisher, the editors and the reviewers. Any product that may be evaluated in this article, or claim that may be made by its manufacturer, is not guaranteed or endorsed by the publisher.
