# Supplementary material for: Epidemiology of malaria and anemia in high and low malaria-endemic North-Eastern districts of India
Source: Front Public Health. 2022 Jul 28;10:940898. doi: 10.3389/fpubh.2022.940898 (PMC9366887; doi:10.3389/fpubh.2022.940898)
Supplement: Supplementary file 1 [file Data_Sheet_1.docx]

**Supplementary Tables**

Supplementary Table 1 – Village-wise population demographics, malaria and anaemia prevalence.

| **DISTRICT** | **BLOCK** | **CHC/PHC** | **VILLAGE** | **ENDEMICITY** | | **AVG. AGE (yrs)** | **MALE/ FEMALE RATIO** | **AVG. Hb** | **FEVER %** | **% ANAEMIA** | **% MALARIA POSITIVE (RDT)** | **% ASYMPTOMATIC CASES** |
| --- | --- | --- | --- | --- | --- | --- | --- | --- | --- | --- | --- | --- |
| **East Garo Hills** | **Rongjeng** | **Rongjeng** | Baringgre Bolkreth | High | | 17.06 | 0.55 | 11.58 | 1.96 | 43.79 | 20.26 | 20.26 |
|  |  |  | Baringrre Imsemdrop | High | | 13.15 | 1.00 | 11.43 | 1.61 | 53.23 | 37.10 | 37.10 |
|  |  |  | Dambo Gitong | High | | 20.28 | 0.48 | 11.46 | 0.00 | 48.92 | 35.25 | 33.09 |
|  |  |  | Dangbo Rangbeng | High | | 18.84 | 0.29 | 11.47 | 6.36 | 40.00 | 6.36 | 4.55 |
|  |  |  | Darugre Sangma | High | | 15.56 | 0.60 |  | 6.93 | 0.00 | 0.00 | 0.00 |
|  |  |  | Doget Purang | High | | 14.35 | 0.78 | 11.64 | 9.09 | 53.03 | 6.06 | 6.06 |
|  |  |  | Dombo Rongjeng | High | | 9.62 | 1.27 | 10.75 | 2.70 | 9.91 | 9.91 | 8.11 |
|  |  |  | Mjolgre Wancho | High | | 24.03 | 0.54 | 10.94 | 7.50 | 70.00 | 35.00 | 30.00 |
|  |  |  | Narangre | High | | 26.55 | 0.31 | 10.96 | 0.00 | 76.47 | 0.00 | 0.00 |
|  |  |  | Nongchram | High | | 9.69 | 0.93 | 12.01 | 34.94 | 37.35 | 16.87 | 7.23 |
|  |  |  | Rongchek Akong | High | | 19.42 | 0.66 | 11.49 | 2.59 | 59.48 | 21.55 | 20.69 |
|  |  |  | Rongdu rongtit | High | | 10.61 | 1.00 | 11.92 | 34.21 | 40.79 | 78.95 | 50.00 |
|  |  |  | Rongdudabith | High | | 19.97 | 1.55 | 12.52 | 22.97 | 31.08 | 22.97 | 13.51 |
|  |  |  | Rongdu-inbol | High | | 13.46 | 0.59 | 11.70 | 52.31 | 44.62 | 27.69 | 9.23 |
|  |  |  | Rongdurangra | High | | 19.26 | 1.19 | 11.81 | 22.03 | 47.46 | 3.39 | 3.39 |
|  |  |  | Rongom | High | | 17.57 | 0.82 |  | 21.57 | 0.00 | 15.69 | 9.80 |
|  |  |  | Tesokgittim | High | | 15.33 | 0.61 | 11.82 | 33.96 | 49.06 | 15.09 | 3.77 |
|  |  |  | Woncho | High | | 13.41 | 0.42 | 10.74 | 3.70 | 55.56 | 37.04 | 33.33 |
|  | **Songsak** | **Songsak** | Asil Songma | Low | | 20.24 | 0.59 | 11.51 | 11.49 | 52.03 | 11.49 | 9.46 |
|  |  |  | Asildikkagre | Low | | 20.16 | 0.36 | 10.84 | 7.14 | 58.16 | 7.14 | 6.12 |
|  |  |  | Barikgre | Low | | 16.09 | 1.21 | 12.14 | 0.00 | 37.10 | 1.61 | 1.61 |
|  |  |  | Bonechisogre | Low | | 33.35 | 1.00 | 12.16 | 0.00 | 50.00 | 1.67 | 1.67 |
|  |  |  | Jamge Songgitcham | Low | | 15.96 | 0.56 | 11.67 | 0.50 | 45.27 | 13.93 | 13.93 |
|  |  |  | Koksi Nengsath | Low | | 24.97 | 0.40 | 11.07 | 4.08 | 63.27 | 4.08 | 4.08 |
|  |  |  | Nakpak apal | Low | | 20.29 | 0.78 | 12.39 | 1.56 | 31.25 | 12.50 | 12.50 |
|  |  |  | Napak Bolchugre | Low | | 21.23 | 1.08 | 11.71 | 0.94 | 58.49 | 21.70 | 21.70 |
|  |  |  | Norek Bolonggre | Low | | 23.46 | 0.85 | 11.64 | 11.22 | 57.14 | 18.37 | 17.35 |
|  |  |  | Rongapgre | Low | | 14.99 | 0.69 | 11.15 | 1.10 | 37.36 | 49.45 | 36.26 |
|  |  |  | Snal Bollong | Low | | 14.28 | 0.68 | 11.95 | 2.44 | 35.77 | 17.89 | 17.07 |
|  |  |  | Songsak wagopgre | Low | | 8.03 | 1.24 | 11.94 | 3.95 | 32.89 | 23.68 | 21.05 |
|  |  |  | Sonsak Market | Low | | 31.68 | 2.09 | 12.96 | 2.94 | 41.18 | 0.00 | 0.00 |
|  |  |  | Sonsak Wagopre | Low | | 26.41 | 0.24 | 11.32 | 0.00 | 65.85 | 7.32 | 7.32 |
|  |  |  | Table Bonegre | Low | | 13.04 | 0.65 | 12.12 | 9.40 | 38.35 | 1.50 | 1.50 |
|  |  |  | Tebilboregre | Low | | 17.18 | 0.00 | 11.89 | 14.29 | 46.43 | 3.57 | 3.57 |
| **Udalguri** | **Bhergaon** | **Khoirabari** | Banargaon | Low | | 20.67 | 0.70 | 11.13 | 0.00 | 69.64 | 0.00 | 0.00 |
|  |  |  | Bhergaon Guest House | Low | | 36.18 | 3.07 | 13.03 | 0.00 | 38.60 | 0.00 | 0.00 |
|  |  |  | Jalukbari | Low | | 13.31 | 0.91 | 11.39 | 17.44 | 54.65 | 0.00 | 0.00 |
|  |  |  | Kharengi | Low | | 25.11 | 1.64 | 11.99 | 4.55 | 50.00 | 0.00 | 0.00 |
|  |  |  | Naobandha | Low | | 16.90 | 1.10 | 12.67 | 14.20 | 20.99 | 0.00 | 0.00 |
|  |  |  | No.3 Amguri | Low | | 24.32 | 0.61 | 11.79 | 8.86 | 54.43 | 0.00 | 0.00 |
|  |  |  | Purani Bhergaon | Low | | 23.29 | 0.75 | 12.14 | 12.09 | 39.56 | 0.00 | 0.00 |
|  | **Khoirabari** | **Khoirabari** | 1 Bholatar | Low | | 28.00 | 0.29 | 11.52 | 0.00 | 59.09 | 0.00 | 0.00 |
|  |  |  | Attareekhat TE | Low | | 34.90 | 2.59 | 13.03 | 0.00 | 36.54 | 0.00 | 0.00 |
|  |  |  | Babrikhat | Low | | 39.11 | 0.18 | 11.58 | 2.13 | 65.96 | 0.00 | 0.00 |
|  |  |  | Badlapara TE | Low | | 13.56 | 0.88 | 11.15 | 0.79 | 58.73 | 0.00 | 0.00 |
|  |  |  | Bamunjuli T.E. | Low | | 14.99 | 0.88 | 12.21 | 0.00 | 36.81 | 0.00 | 0.00 |
|  |  |  | Batabari | Low | | 17.45 | 0.45 | 11.79 | 0.00 | 49.22 | 0.00 | 0.00 |
|  |  |  | Batabari L. P. School | Low | | 11.00 | 1.80 | 11.72 | 0.00 | 46.43 | 0.00 | 0.00 |
|  |  |  | Borungujuli | Low | | 14.31 | 0.80 | 11.50 | 10.16 | 50.00 | 0.00 | 0.00 |
|  |  |  | Chewni | Low | | 14.92 | 0.82 | 12.17 | 0.00 | 39.69 | 0.00 | 0.00 |
|  |  |  | Darogakhat | Low | | 42.28 | 0.67 | 11.49 | 2.50 | 77.50 | 0.00 | 0.00 |
|  |  |  | Dongpara | Low | | 28.45 | 0.51 | 11.32 | 0.00 | 62.26 | 0.00 | 0.00 |
|  |  |  | Geruajhar | Low | | 16.15 | 0.47 | 12.27 | 0.00 | 31.58 | 0.00 | 0.00 |
|  |  |  | Katara | Low | | 28.85 | 0.83 | 11.97 | 0.00 | 53.28 | 0.00 | 0.00 |
|  |  |  | Khagra | Low | | 30.23 | 0.71 | 11.66 | 0.00 | 57.66 | 0.00 | 0.00 |
|  |  |  | Khoiragrant | Low | | 19.75 | 0.84 | 12.21 | 0.00 | 29.21 | 0.00 | 0.00 |
|  |  |  | Nanaipara TE | Low | | 11.07 | 0.94 | 11.50 | 14.14 | 50.51 | 0.51 | 0.00 |
|  |  |  | Nisolabari | Low | | 20.00 | 0.48 | 10.27 | 10.74 | 80.17 | 0.00 | 0.00 |
|  |  |  | No.4 Rajagarh | Low | | 20.37 | 1.10 | 12.06 | 13.64 | 42.42 | 0.00 | 0.00 |
|  |  |  | Nonai khuti | Low | | 12.08 | 1.60 | 10.67 | 8.24 | 76.37 | 0.00 | 0.00 |
|  |  |  | Panisheli | Low | | 18.88 | 0.65 | 11.73 | 0.68 | 53.74 | 0.00 | 0.00 |
|  |  |  | Rangagaon | Low | | 29.05 | 0.54 | 11.71 | 6.56 | 56.56 | 0.00 | 0.00 |
|  |  |  | Tamrewsar | Low | | 15.45 | 0.70 | 10.98 | 0.00 | 70.97 | 0.00 | 0.00 |
|  |  |  | Tenkibasti | Low | | 16.74 | 0.82 | 11.66 | 15.89 | 54.97 | 0.00 | 0.00 |
|  | **Orang** | **Mazbat** | 10 No Basti | High | | 16.67 | 0.89 | 9.67 | 0.00 | 95.51 | 6.74 | 6.74 |
|  |  |  | 2 No Bagaribari | High | | 20.87 | 0.37 | 10.11 | 0.00 | 88.06 | 2.99 | 2.99 |
|  |  |  | 24 No line | High | | 18.95 | 0.65 | 10.09 | 0.00 | 92.86 | 8.93 | 8.93 |
|  |  |  | Bahipukhuri 3 | High | | 21.36 | 0.49 |  | 0.60 | 0.00 | 0.00 | 0.00 |
|  |  |  | Bahipukri Noline | High | | 20.05 | 0.52 | 10.88 | 0.00 | 63.83 | 0.00 | 0.00 |
|  |  |  | Dalanibasti | High | | 14.62 | 1.00 | 10.71 | 2.59 | 76.72 | 4.31 | 4.31 |
|  |  |  | Dhanseni Hospital | High | | 23.48 | 0.63 | 10.67 | 14.04 | 77.19 | 5.26 | 3.51 |
|  |  |  | Dhunseni 6 Noline | High | | 13.41 | 0.52 | 10.54 | 0.00 | 83.56 | 13.70 | 13.70 |
|  |  |  | Dhunseri No 3 | High | | 18.66 | 0.51 | 11.25 | 7.69 | 64.62 | 4.62 | 3.08 |
|  |  |  | Dimarung Line | High | | 14.41 | 0.63 | 10.86 | 5.41 | 74.77 | 5.41 | 5.41 |
|  |  |  | Dimasang | High | | 24.32 | 0.47 | 10.63 | 1.52 | 53.03 | 1.52 | 1.52 |
|  |  |  | Khiyajnli | High | | 19.53 | 0.93 | 11.07 | 3.45 | 48.28 | 1.15 | 1.15 |
|  |  |  | Kukurphuka | High | | 26.79 | 0.57 | 11.24 | 0.00 | 58.33 | 0.00 | 0.00 |
|  |  |  | Lalpani | High | | 14.17 | 0.95 | 11.32 | 1.16 | 58.14 | 3.49 | 3.49 |
|  |  |  | Monai No 1 | High | | 17.03 | 0.95 | 10.59 | 2.63 | 75.00 | 6.58 | 6.58 |
|  |  |  | Munaigaon No.2 | High | | 19.91 | 0.74 | 11.32 | 1.37 | 50.68 | 9.59 | 9.59 |
|  |  |  | Nagaon | High | | 22.52 | 0.50 | 11.16 | 0.00 | 72.73 | 4.55 | 4.55 |
|  |  |  | Naoherua (N) | High | | 16.05 | 0.87 | 11.55 | 10.43 | 60.66 | 0.00 | 0.00 |
|  |  |  | Nizrangapani | High | | 28.53 | 0.70 | 12.49 | 6.52 | 38.04 | 0.00 | 0.00 |
|  |  |  | No.1 Rangapani | High | | 18.99 | 0.64 | 11.16 | 0.00 | 66.67 | 0.00 | 0.00 |
|  |  |  | No.1 Thakurpar | High | | 26.42 | 0.33 | 10.56 | 8.33 | 75.00 | 0.00 | 0.00 |
|  |  |  | No.3 Rangapai | High | | 15.98 | 1.21 | 11.60 | 4.76 | 52.38 | 0.00 | 0.00 |
|  |  |  | No.4 Rangapani | High | | 8.40 | 1.00 | 11.26 | 10.42 | 54.17 | 0.00 | 0.00 |
|  |  |  | Paharpur | High | | 23.89 | 0.56 | 11.18 | 10.71 | 62.50 | 3.57 | 3.57 |
|  |  |  | Pahartuli | High | | 21.44 | 1.00 | 10.56 | 3.33 | 71.67 | 6.67 | 6.67 |
|  |  |  | Pahartuli 19 No | High | | 16.88 | 1.13 | 10.69 | 2.94 | 76.47 | 0.00 | 0.00 |
|  |  |  | Pathahpur | High | | 20.20 | 0.60 | 11.28 | 16.24 | 58.12 | 0.00 | 0.00 |
|  |  |  | Pathani Lina | High | | 13.54 | 0.82 |  | 1.25 | 0.00 | 2.50 | 2.50 |
|  |  |  | Routa Habi Gaon | High | | 24.92 | 1.78 | 10.70 | 0.00 | 88.00 | 0.00 | 0.00 |
|  |  |  | Sikaridanga No.2 | High | | 17.00 | 0.78 | 10.96 | 4.85 | 46.60 | 0.97 | 0.97 |
|  |  | **Orang** | Dhunserio Line no. 7 | High | | 14.43 | 0.92 | 10.93 | 0.98 | 69.61 | 1.96 | 1.96 |
| **Grand Total** | | | | |  | 18.69 | 0.74 | 11.55 | 6.08 | 49.84 | 6.54 | 5.52 |

*Supplementary Table 2: Village wise stratification of Anaemia in high (East Garo Hills) and low (Udalguri) endemic districts*

| **District** | **Block** | **Village** | **No Anaemia** | **Mild Anaemia** | **Moderate Anaemia** | **Severe Anaemia** | **Grand Total** |
| --- | --- | --- | --- | --- | --- | --- | --- |
| **East Garo Hills** | **Rongjeng** | Baringgre Bolkreth | 58 | 35 | 31 | 1 | **125** |
|  |  | Baringrre Imsemdrop | 29 | 5 | 27 | 1 | **62** |
|  |  | Dambo Gitong | 64 | 26 | 39 | 3 | **132** |
|  |  | Dangbo Rangbeng | 38 | 23 | 18 | 3 | **82** |
|  |  | Darugre Sangma |  |  |  |  |  |
|  |  | Doget Purang | 30 | 18 | 16 | 1 | **65** |
|  |  | Dombo Rongjeng | 4 | 5 | 6 |  | **15** |
|  |  | Mjolgre Wancho | 12 | 10 | 16 | 2 | **40** |
|  |  | Narangre | 3 | 6 | 7 |  | **16** |
|  |  | Nongchram | 52 | 17 | 14 |  | **83** |
|  |  | Rongchek Akong | 88 | 51 | 85 | 2 | **226** |
|  |  | Rongdu rongtit | 44 | 22 | 9 |  | **75** |
|  |  | Rongdudabith | 49 | 15 | 7 | 1 | **72** |
|  |  | Rongdu-inbol | 36 | 16 | 13 |  | **65** |
|  |  | Rongdurangra | 31 | 17 | 11 |  | **59** |
|  |  | Rongom |  |  |  |  |  |
|  |  | Tesokgittim | 27 | 14 | 12 |  | **53** |
|  |  | Woncho | 11 | 3 | 10 | 2 | **26** |
|  |  | **Total** | **576** | **283** | **321** | **16** | **1196** |
|  | **Songsak** | Asil Songma | 71 | 39 | 35 | 3 | **148** |
|  |  | Asildikkagre | 41 | 14 | 40 | 3 | **98** |
|  |  | Barikgre | 39 | 12 | 10 | 1 | **62** |
|  |  | Bonechisogre | 30 | 17 | 12 | 1 | **60** |
|  |  | Jamge Songgitcham | 110 | 46 | 41 | 4 | **201** |
|  |  | Koksi Nengsath | 18 | 15 | 13 | 3 | **49** |
|  |  | Nakpak apal | 44 | 13 | 7 |  | **64** |
|  |  | Napak Bolchugre | 44 | 32 | 27 | 3 | **106** |
|  |  | Norek Bolonggre | 42 | 28 | 28 |  | **98** |
|  |  | Rongapgre | 22 | 13 | 21 |  | **56** |
|  |  | Snal Bollong | 77 | 17 | 25 | 2 | **121** |
|  |  | Songsak wagopgre | 51 | 10 | 15 |  | **76** |
|  |  | Sonsak Market | 20 | 8 | 5 | 1 | **34** |
|  |  | Sonsak Wagopre | 14 | 14 | 13 |  | **41** |
|  |  | Table Bonegre | 164 | 53 | 46 | 3 | **266** |
|  |  | Tebilboregre | 15 | 7 | 6 |  | **28** |
|  |  | **Total** | **802** | **338** | **344** | **24** | **1508** |
| **Udalguri** | **Bhergaon** | Banargaon | 34 | 32 | 44 | 2 | **112** |
|  |  | Bhergaon Guest House | 35 | 17 | 4 | 1 | **57** |
|  |  | Jalukbari | 39 | 17 | 30 |  | **86** |
|  |  | Kharengi | 33 | 18 | 15 |  | **66** |
|  |  | Naobandha | 128 | 25 | 7 | 2 | **162** |
|  |  | No.3 Amguri | 36 | 25 | 18 |  | **79** |
|  |  | Purani Bhergaon | 54 | 21 | 15 |  | **90** |
|  |  |  | **359** | **155** | **133** | **5** | **652** |
|  | **Khoirabari** | 1 Bholatar | 9 | 5 | 8 |  | **22** |
|  |  | Attareekhat TE | 62 | 27 | 8 | 3 | **100** |
|  |  | Babrikhat | 16 | 15 | 15 | 1 | **47** |
|  |  | Badlapara TE | 52 | 30 | 38 | 6 | **126** |
|  |  | Bamunjuli T.E. | 115 | 38 | 28 | 1 | **182** |
|  |  | Batabari | 65 | 33 | 30 |  | **128** |
|  |  | Batabari L. P. School | 15 | 7 | 6 |  | **28** |
|  |  | Borungujuli | 64 | 29 | 31 | 4 | **128** |
|  |  | Chewni | 79 | 32 | 19 | 1 | **131** |
|  |  | Darogakhat | 9 | 18 | 13 |  | **40** |
|  |  | Dongpara | 40 | 31 | 35 |  | **106** |
|  |  | Geruajhar | 130 | 33 | 27 |  | **190** |
|  |  | Katara | 64 | 44 | 28 | 1 | **137** |
|  |  | Khagra | 58 | 40 | 38 | 1 | **137** |
|  |  | Khoiragrant | 111 | 24 | 34 | 1 | **170** |
|  |  | Nanaipara TE | 98 | 47 | 50 | 3 | **198** |
|  |  | Nisolabari | 8 | 19 | 77 | 1 | **105** |
|  |  | No.4 Rajagarh | 76 | 35 | 21 |  | **132** |
|  |  | Nonai khuti | 43 | 44 | 95 |  | **182** |
|  |  | Panisheli | 68 | 40 | 39 |  | **147** |
|  |  | Rangagaon | 53 | 35 | 34 |  | **122** |
|  |  | Tamrewsar | 36 | 30 | 57 | 1 | **124** |
|  |  | Tenkibasti | 68 | 50 | 29 | 4 | **151** |
|  |  | **Total** | **1339** | **706** | **760** | **28** | **2833** |
|  | **Orang** | 10 No Basti | 3 | 22 | 55 | 8 | **88** |
|  |  | 2 No Bagaribari | 8 | 13 | 42 | 4 | **67** |
|  |  | 24 No line | 4 | 12 | 37 | 3 | **56** |
|  |  | Bahipukhuri 3 |  |  |  |  |  |
|  |  | Bahipukri Noline | 13 | 28 | 30 | 2 | **73** |
|  |  | Dalanibasti | 27 | 31 | 54 | 4 | **116** |
|  |  | Dhanseni Hospital | 13 | 16 | 22 | 6 | **57** |
|  |  | Dhunseni 6 Noline | 10 | 19 | 40 | 2 | **71** |
|  |  | Dhunseri No 3 | 23 | 18 | 24 |  | **65** |
|  |  | Dimarung Line | 28 | 37 | 45 | 1 | **111** |
|  |  | Dimasang | 8 | 11 | 23 | 1 | **43** |
|  |  | Khiyajnli | 24 | 15 | 26 | 1 | **66** |
|  |  | Kukurphuka | 15 | 8 | 12 | 1 | **36** |
|  |  | Lalpani | 36 | 28 | 21 | 1 | **86** |
|  |  | Monai No 1 | 19 | 17 | 35 | 5 | **76** |
|  |  | Munaigaon No.2 | 36 | 11 | 23 | 3 | **73** |
|  |  | Nagaon | 18 | 24 | 24 |  | **66** |
|  |  | Naoherua (N) | 82 | 68 | 58 | 2 | **210** |
|  |  | Nizrangapani | 57 | 24 | 11 |  | **92** |
|  |  | No.1 Rangapani | 12 | 10 | 14 |  | **36** |
|  |  | No.1 Thakurpar | 2 | 8 | 8 | 2 | **20** |
|  |  | No.3 Rangapai | 20 | 8 | 14 |  | **42** |
|  |  | No.4 Rangapani | 22 | 10 | 16 |  | **48** |
|  |  | Paharpur | 21 | 13 | 20 | 2 | **56** |
|  |  | Pahartuli | 7 | 16 | 26 | 1 | **50** |
|  |  | Pahartuli 19 No | 8 | 11 | 13 | 2 | **34** |
|  |  | Pathahpur | 40 | 34 | 32 | 2 | **108** |
|  |  | Pathani Lina |  |  |  |  |  |
|  |  | Routa Habi Gaon | 3 | 9 | 12 | 1 | **25** |
|  |  | Sikaridanga No.2 | 18 | 19 | 29 |  | **66** |
|  |  | Dhunserio Line no. 7 | 31 | 26 | 43 | 2 | **102** |
|  |  | **Total** | **608** | **566** | **809** | **56** | **2039** |
| **Grand Total** |  |  | **3684** | **2048** | **2367** | **129** | **8228** |
